# Supplementary material for: Targeted Time‐Varying Functional Connectivity
Source: Hum Brain Mapp. 2025 Mar 4;46(4):e70157. doi: 10.1002/hbm.70157 (PMC11876989; doi:10.1002/hbm.70157)
Supplement: Supplementary file 1 — Figure S1. Similarity scores across 100 repetitions of the T‐TVFC and the conventional (covariance based) HMM. Figure S2. Percentage of explained variance by the first principal component for the 11 cortical networks and two thalamic nuclei (AL and Pu). PCA was not applied to MD nuclei as it consists of only one region. Individual scores represent trial‐averaged for each subject and per condition (binary, ternary and quaternary). Boxes represent the standard deviation, lighter shading indicates the standard error, and the dashed line denotes the mean across subjects. * indicates pairwise differences with FWEcorrected p‐value < 0.05, obtained through non‐parametric (n = 5000) permutation testing. AL: Anterolateral thalamic nuclei; AUD: Auditory; CP: Cinguloparietal; CO: Cingulopercular; DM: Default Mode; DAT: Dorsal Attention; FP: Frontoparietal; MD: Medial Dorsal thalamic nuclei; Pu: Pulvinar thalamic nuclei; RT: Retrosplenial Temporal; SAL: Salience; SM: Sensorimotor; VAT: Ventral Attention; VIS: Visual. Figure S3. Mean (± SD) trial‐locked time series across subjects from 11 cortical networks and three thalamic nuclei under binary, ternary and quaternary conditions. AL: Anterolateral thalamic nuclei; AUD: Auditory; CP: Cinguloparietal; CO: Cingulopercular; DM: Default Mode; DAT: Dorsal Attention; FP: Frontoparietal; MD: Medial Dorsal thalamic nuclei; Pu: Pulvinar thalamic nuclei; RT: Retrosplenial Temporal; SAL: Salience; SM: Sensorimotor; VAT: Ventral Attention; VIS: Visual. Figure S4. Signal‐to‐noise ratio (SNR) for the 11 cortical network timeseries and three thalamic nuclei timeseries. Individual scores represent trial‐averaged for each subject and per condition (binary, ternary and quaternary). Boxes represent the standard deviation, lighter shading indicates the standard error, and the dashed line denotes the mean across subjects. AL: Anterolateral thalamic nuclei; AUD: Auditory; CP: Cinguloparietal; CO: Cingulopercular; DM: Default Mode; DAT: Dorsal Attentio [file HBM-46-e70157-s001.pdf]

Supplementary Material

corresponding to article:

Targeted Time-Varying Functional Connectivity

Sonsoles Alonso, Luca Cocchi, Luke J. Hearne, James M. Shine and Diego Vidaurre

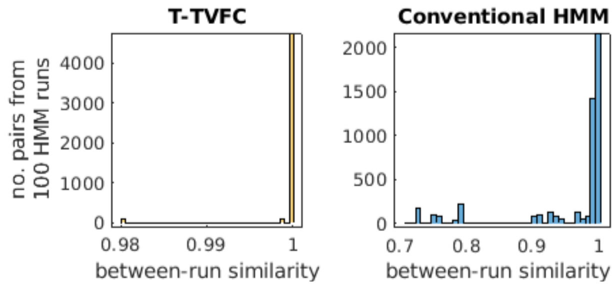

Figure S1. Similarity scores across 100 repetitions of the T-TVFC and the conventional (covariance-based) HMM.

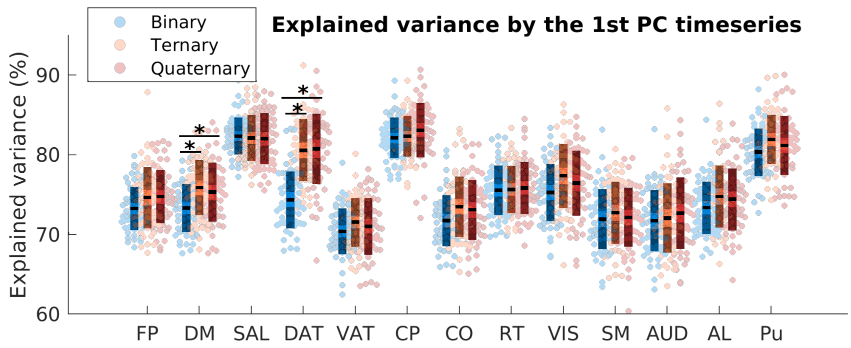

Figure S2. Percentage of explained variance by the first principal component for the 11 cortical networks and two thalamic nuclei (AL and Pu). PCA was not applied to MD nuclei as it consists of only one region. Individual scores represent trial-averaged for each subject and per condition (binary, ternary and quaternary). Boxes represent the standard deviation, lighter shading indicates the standard error, and the dashed line denotes the mean across subjects. * indicates pairwise differences with FWE-corrected p-values < 0.05, obtained through non-parametric ($n=5000$) permutation testing. Abbreviations: AL: Anterolateral thalamic nuclei; AUD: Auditory; CP: Cinguloparietal; CO: Cingulopercular; DM: Default Mode; DAT: Dorsal Attention; FP: Frontoparietal; MD: Medial Dorsal thalamic nuclei; Pu: Pulvinar thalamic nuclei; RT: Retrosplenial Temporal; SAL: Salience; SM: Sensorimotor; VAT: Ventral Attention; VIS: Visual.

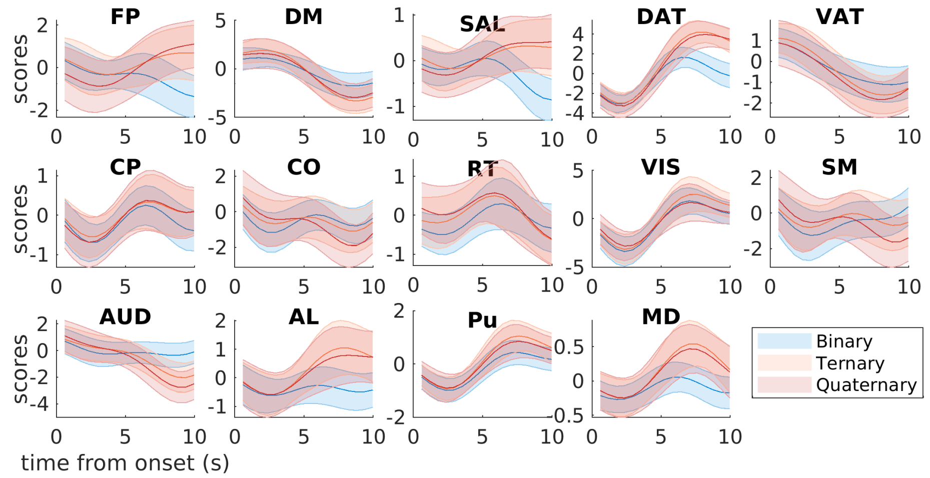

Figure S3. Mean (± SD) trial-locked time series across subjects from 11 cortical networks and three thalamic nuclei under binary, ternary and quaternary conditions. Abbreviations: AL: Anterolateral thalamic nuclei; AUD: Auditory; CP: Cinguloparietal; CO: Cingulopercular; DM: Default Mode; DAT: Dorsal Attention; FP: Frontoparietal; MD: Medial Dorsal thalamic nuclei; Pu: Pulvinar thalamic nuclei; RT: Retrosplenial Temporal; SAL: Salience; SM: Sensorimotor; VAT: Ventral Attention; VIS: Visual.

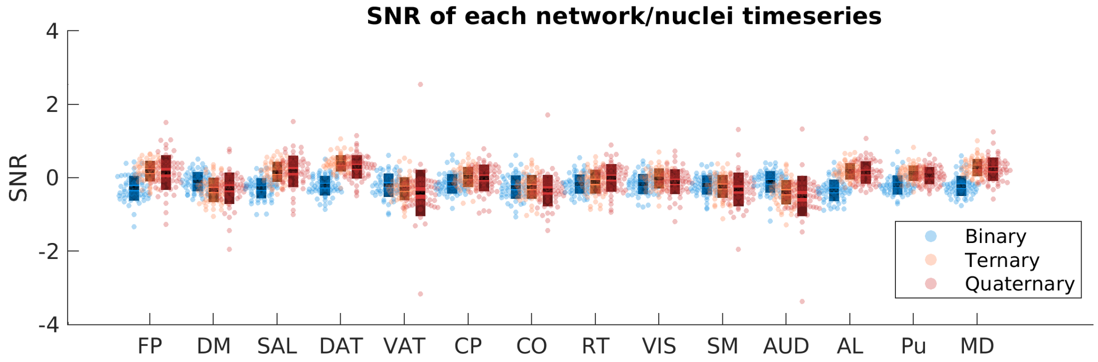

Figure S4. Signal-to-noise ratio (SNR) for the 11 cortical network timeseries and three thalamic nuclei timeseries. Individual scores represent trial-averaged for each subject and per condition (binary, ternary and quaternary). Boxes represent the standard deviation, lighter shading indicates the standard error, and the dashed line denotes the mean across subjects. Abbreviations: AL: Anterolateral thalamic nuclei; AUD: Auditory; CP: Cinguloparietal; CO: Cingulopercular; DM: Default Mode; DAT: Dorsal Attention; FP: Frontoparietal; MD: Medial Dorsal thalamic nuclei; Pu: Pulvinar thalamic nuclei; RT: Retrosplenial Temporal; SAL: Salience; SM: Sensorimotor; VAT: Ventral Attention; VIS: Visual.

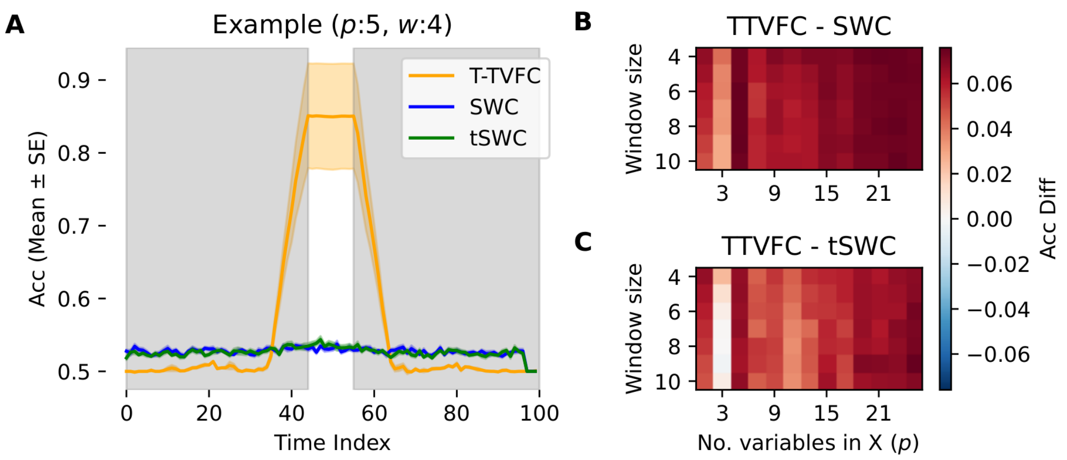

Figure S5. Model performance compared to SWC approaches using simulated data. **A**. Example illustrating the performance of the T-TVFC (orange) compared to all-pairs SWC (in blue) and specific-pairs SWC (tSWC, in green) over time for a specific parameter configuration ($p = 5$, $\sigma = 0.1$ and $w = 4$). Accuracy in predicting trial conditions, represented as the mean ± standard error over 10 repetitions, was calculated using logistic regression and evaluated against the chance level of 0.5. The white patch indicates the period where the simulated task-dependent state was active. **B**. Differences in mean accuracies between T-TVFC and all-pairs SWC. **C**. Differences in mean accuracies between T-TVFC and specific-pairs SWC, across varying values of p and w. Mean accuracies for each model were computed during the interval when there are differences between conditions. p: number of predictors in X. σ: levels of noise. w: window size used in SWC.

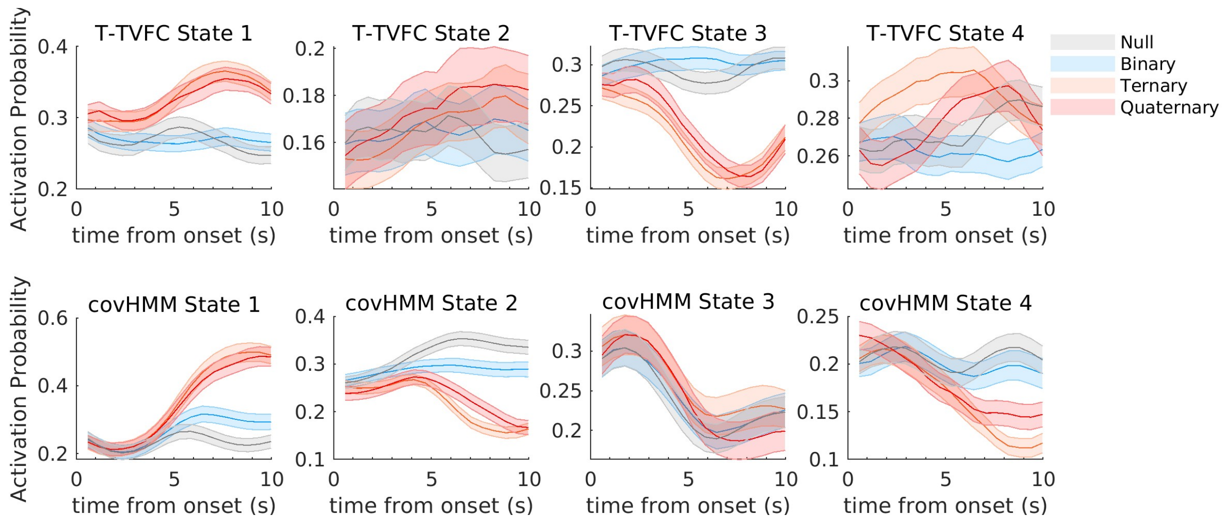

Figure S6. Trial-locked state time courses from T-TVFC (*top*) and covariance-based HMM (*bottom*), both configured with $K = 4$ states. Increasing the number of states from $K = 3$ to $K = 4$ does not provide additional insights but instead divides existing states into more granular representations.

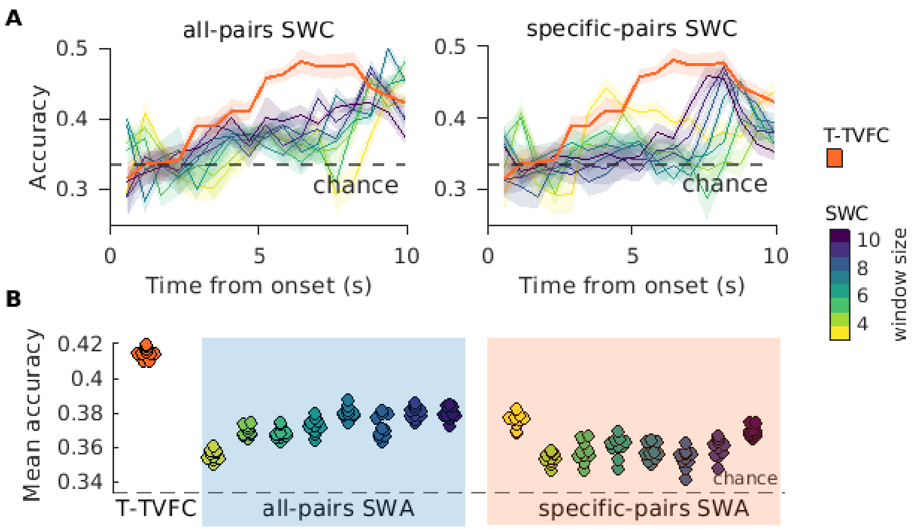

Figure S7. Model performance compared to SWC approaches using real data. **A.** Classification accuracy of T-TVFC (orange) compared to all-pairs SWC (left) and specific-pairs SWC (right) across a range of window sizes (3 to 10 TRs; see color bar). Accuracy values, represented as the mean and standard deviation over 10 repetitions, were obtained using ordinal logistic regression and evaluated at different levels of task complexity (binary, ternary, and quaternary classification). Results are compared against the 1/3 chance level. **B**. Average classification accuracy over the 10-second trial duration for T-TVFC (orange) compared to all-pairs SWC (blue shading) and specific-pairs SWC (orange shading), evaluated across varying window sizes.
